# Change of hypothalamic adult neurogenesis in mice by chronic treatment of fluoxetine

**DOI:** 10.1186/s13104-022-05954-z

**Published:** 2022-02-16

**Authors:** Koji Ohira

**Affiliations:** Laboratory of Nutritional Brain Science, Department of Food Science and Nutrition, Mukogawa Women’s University, 6-46 Ikebiraki, Nishinomiya, Hyogo 663-8558 Japan

**Keywords:** Adult neurogenesis, Antidepressant, Hypothalamus, Neural stem cell

## Abstract

**Objective:**

More than half of patients with depression display eating disorders, such as bulimia nervosa and anorexia nervosa. Feeding centers are located in the hypothalamus, and hypothalamic adult neurogenesis has an important role in feeding and energy balance. Antidepressants, which can regulate adult neurogenesis in the hippocampus, olfactory bulb, and neocortex, are used for eating disorders, but it is unclear whether antidepressants change hypothalamic adult neurogenesis. In this study, we used immunohistological analysis to assess effects of the antidepressant fluoxetine (FLX) on hypothalamic adult neurogenesis of adult mice.

**Results:**

Expressions of the proliferating cell marker, Ki67, and the neural stem cell marker, nestin, were significantly decreased in the hypothalamus by FLX. As regard to postmitotic cells, the number of the neural marker, NeuN, positive cells was significantly upregulated by FLX, but that of the astrocytic marker, S100B, positive cells was significantly reduced by FLX. The number of the oligodendrocyte marker, Olig2, positive cells was not changed by FLX. Interestingly, FLX treatment did not affect the total number of newly generated cells in the hypothalamus, comparing that in controls. These results suggest that FLX treatment influence hypothalamic adult neurogenesis and shift the balance between the numbers of neurons and astrocytes under studied conditions.

**Supplementary Information:**

The online version contains supplementary material available at 10.1186/s13104-022-05954-z.

## Introduction

Many studies have demonstrated that new neurons are generated in limited regions of the adult brain, such as the hippocampal dentate gyrus (HDG), subventricular zone (SVZ), cerebral cortex, and hypothalamus [1–3]. Neural stem cells (NSCs), which can produce neuronal progenitor cells (NPCs) and glial progenitor cells, and each progenitor cell, which can produce postmitotic neurons and glial cells, are found in these regions, and can persist neurogenesis under physiological conditions, throughout life [4–6]. Although it is still controversial whether sufficient numbers of neurons are produced in these regions of adult humans [7–9], adult neurogenesis, NSCs, and NPCs are present in primates, including humans [10–12]. It is becoming increasingly clear that new neurons in the existing neural network may function in adult brains. Neurogenesis in the HDG and SVZ is related to the memory of events and odor, respectively [13]. New neurons in the cerebral cortex have neuroprotective function against brain ischemia [14].

In the past 2 decades, evidence has accumulated that neurogenesis can occur in adult mammalian hypothalamus. In the third ventricle of the hypothalamus, NSCs exist and produce new neurons. Hypothalamic NSCs, called tanycytes, are subdivided into four types, such as α1, α2, β1, and β2, based on their position, gene expression pattern, innervation, and neurogenic potential. The α2-tanycytes display neurogenic characteristics. The functions of the hypothalamus are involved in the regulation of metabolism, reproduction, endocrine, sleep, and body temperature, and new neurons in the hypothalamus are expected to be related to those functions. In fact, new neurons contain gonadotropin-releasing hormone, thyrotropin-releasing hormone, oxytocin, and vasopressin [15]. Recent studies have found the role of hypothalamic neurogenesis in metabolic regulation and reproductive physiology [16]. Levels of hypothalamic neurogenesis can be regulated by dietary, environmental and hormonal signals. Since the hypothalamus has a central role in controlling a broad range of homeostatic physiological processes, these findings may have far ranging behavioral and medical implications [17].

The abnormalities of adult neurogenesis in these regions have been reported to be involved in neuropsychiatric disorders, such as Alzheimer’s disease and depression. There is a significant decrease in HDG neurogenesis in patients with Alzheimer’s disease and some animal models [18]. Although there is no direct evidence that adult neurogenesis is decreased in patients with depression, stress, which is one of the major factors for depression, decreases adult neurogenesis in the HDG of experimental animals [19]. In addition, NSCs and NPCs in the HDG, cerebral cortex, and SVZ, are affected by antidepressants. Chronic antidepressant treatment increases neurogenesis in the HDG [20] and cerebral cortex [14], and has the opposite effect on neurogenesis in the SVZ [21].

Depression is a common mental disorder, affecting approximately 300 million people worldwide in 2017 [22]. More than 50% of patients with depression have been reported to show eating disorders [23]. For patients with eating disorders, selective serotonin reuptake inhibitors (SSRIs), a group of antidepressants, are used for drug treatment [24]. Hypothalamic neurogenesis functions as a regulator of eating behaviors and energy balance [25]. However, there are almost no data that SSRIs influence hypothalamic neurogenesis, although early exposure to SSRIs is linked to depression and anxiety-like disorders [26]. In this study, we examined whether the SSRI, fluoxetine (FLX), administration affect hypothalamic neurogenesis in adult mice, using immunohistological methods.

## Main text

### Materials and methods

#### Experimental animals

Eight adult male C57BL/6 J mice (8-week-old; Japan SLC, Shizuoka, Japan) were used in this study. Housing conditions were thermostatically maintained at 24 ± 1 °C with constant humidity (60%) and lighting (12 h light/dark cycle, light on: 7:00–19:00). The animals were housed for 1 week before the experiments and fed a normal diet and water given ad libitum. The experimental procedures for animals were executed in accordance with Mukogawa Women’s University’s guidelines for the ethical treatment of laboratory animal.

#### FLX and bromodeoxyuridine (BrdU) treatments

FLX and BrdU treatments were performed as described previously [1]. Briefly, 8 mice were randomly divided into 2 groups (4 mice/group); each group of mice was intraperitoneally injected with vehicle (phosphate-buffered saline, PBS) or 15 mg/kg FLX (LKT Laboratories, St Paul, MN) every day for 4 weeks. From previous studies [27, 28], assuming a FLX-altered cell volatility of 40–50%, about 4 animals are required per group. In addition, intraperitoneal administration was performed to keep the daily FLX dose was constant. At 1 week after the onset of FLX treatment, an injection of BrdU (100 mg/kg; Sigma Aldrich, St. Louis, MO) was administered at 10 AM once a day for 3 consecutive days. After 4 weeks, the mice were deeply anesthetized with 3% isoflurane (Wako, Osaka, Japan), killed by bloodletting from the right atrium, and perfused with 4% paraformaldehyde (Merck, Darmstadt, Germany) in PBS. The brains were removed and immersed 4% paraformaldehyde overnight at 4 °C. Then, the brains were store in PBS containing 20% sucrose for cryoprotection until use. Body weights of mice were measured every week from the onset of the experiment. The rationale regarding the animals used in the experiment, administration method, and group size is in the Additional file 1.

#### Immunofluorescent staining

Immunofluorescent staining for brain sections was performed as described previously [14]. Briefly, the brains were cut into 50 μm-thick coronal sections using a microtome (LS-113; Yamato Kohki Industrial, Saitama, Japan). Sections were stored in PBS containing sodium azide (0.05%, w/v) at 4 °C until use. Four mice were used to stain sections with each cell type marker antibody; proliferating cells, neurons, astrocytes, and oligodendrocytes.

For BrdU staining, sections were incubated at 4 °C for 10 min in 0.1 N HCl and then at 37 °C for 30 min in 2 N HCl. Sections were washed twice for 5 min in PBS and then blocked in 0.2 M glycine in PBS at RT for at least 2 h. The following procedures were the same as methods with other primary antibodies.

Sections were incubated with primary antibodies overnight at RT. The list of antibodies used in the experiments is given in the Additional file 2. After washing in PBS for 30 min, sections were incubated at RT for 1 h with secondary antibodies. Sections were then washed in PBS for 30 min, mounted on glass slides coated with 3-aminopropyltriethoxysilane, and embedded with PermaFluor (Thermo Fisher Scientific, Waltham, MA, USA).

Images were acquired by using an LSM 510 confocal laser-scanning microscope (Carl Zeiss, Oberkochen, Germany) with a pinhole setting that corresponded to a focal plane thickness of less than 1 μm to obtain images of the stained sections. Quantitative analysis was performed as reported previously [14]. Quantification of positive structures was performed in a blinded manner, i.e., encoded images were assessed in random order by other investigators, although investigators could not be blinded to the groups due to breeding in the difference cages.

We examined the hypothalamic region, which approximately corresponds to − 1.46 to − 2.06 mm posterior from bregma in the atlas of Franklin and Paxinos [29]. Approximately 12 sections from each animal (4 FLX-treated and 4 control mice) were obtained. The sections from each group were divided into 4 groups: for NSCs, for neurons, for astrocytes, and for oligodendrocytes, each of which comprised 12 sections. Positive signals were counted in the whole structures of thalamus.

#### Data analysis

All data are presented as mean  ±  SD. GraphPad Prism (version 6, GraphPad Software, La Jolla, CA) was used to analyze all data. For all statistical analyses used, the alpha level was set at P  < 0.05. Differences between FLX-treated and control groups were compared using unpaired t test. There were no criteria used for including and excluding experimental units.

## Results

**Fig. 1:**
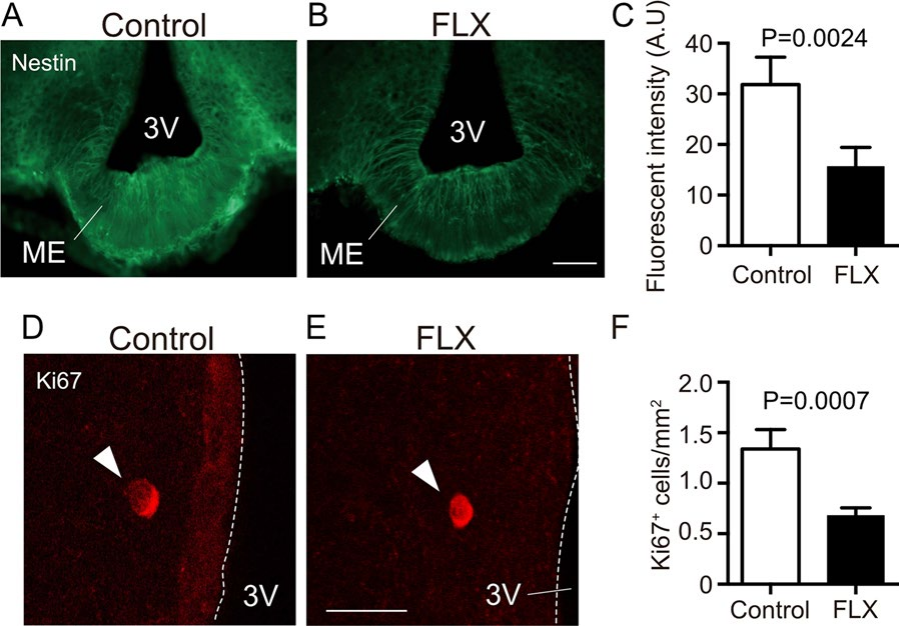
FLX treatment decreased the number of proliferating cells in the hypothalamus of adult mice. **A**, **B** Representative images of the NSC marker, nestin, positive structures in the hypothalamus of mice treated with vehicle (upper) or FLX (lower). **C** The fluorescence intensities of nestin-positive structure were quantified in the hypothalamus of vehicle-treated and FLX-treated mice (n = 4 mice each). **D**, **E** Representative images of the dividing cell marker, Ki67, positive cells in the hypothalamus of mice treated with vehicle (upper) or FLX (lower). **F** The number of Ki67-positive cells was quantified in the hypothalamus of vehicle-treated and FLX-treated mice (n = 4 mice each). Scale bar, 100 μm in **A** and **B**, 20 μm in **D** and **E**. *3V* third ventricle; *ME* medial eminence. Values (mean ± SD) are analyzed by Student’s t test

Hypothalamic NSCs have been reported to express the NSC marker, nestin. Proliferating cells, including NSCs and NPCs, express Ki67 protein in their nuclei. First, by using these markers, we determined whether NSCs and NPCs in the hypothalamus were affected by FLX treatments. The fluorescence intensity of nestin-positive (+) structures was significantly reduced by FLX treatments, compared with controls (P = 0.0024) (Fig. 1A–C). In addition, the number of Ki67^+^ cells was significantly decreased in the hypothalamus of FLX-treated mice, compared with controls (P = 0.0007) (Fig. 1D–F). The proliferation ability of NSCs and NPCs correlates with the level of nestin expression [30]. These results suggest that chronic FLX administration down-regulates the densities of NSCs and NPCs.

**Fig. 2:**
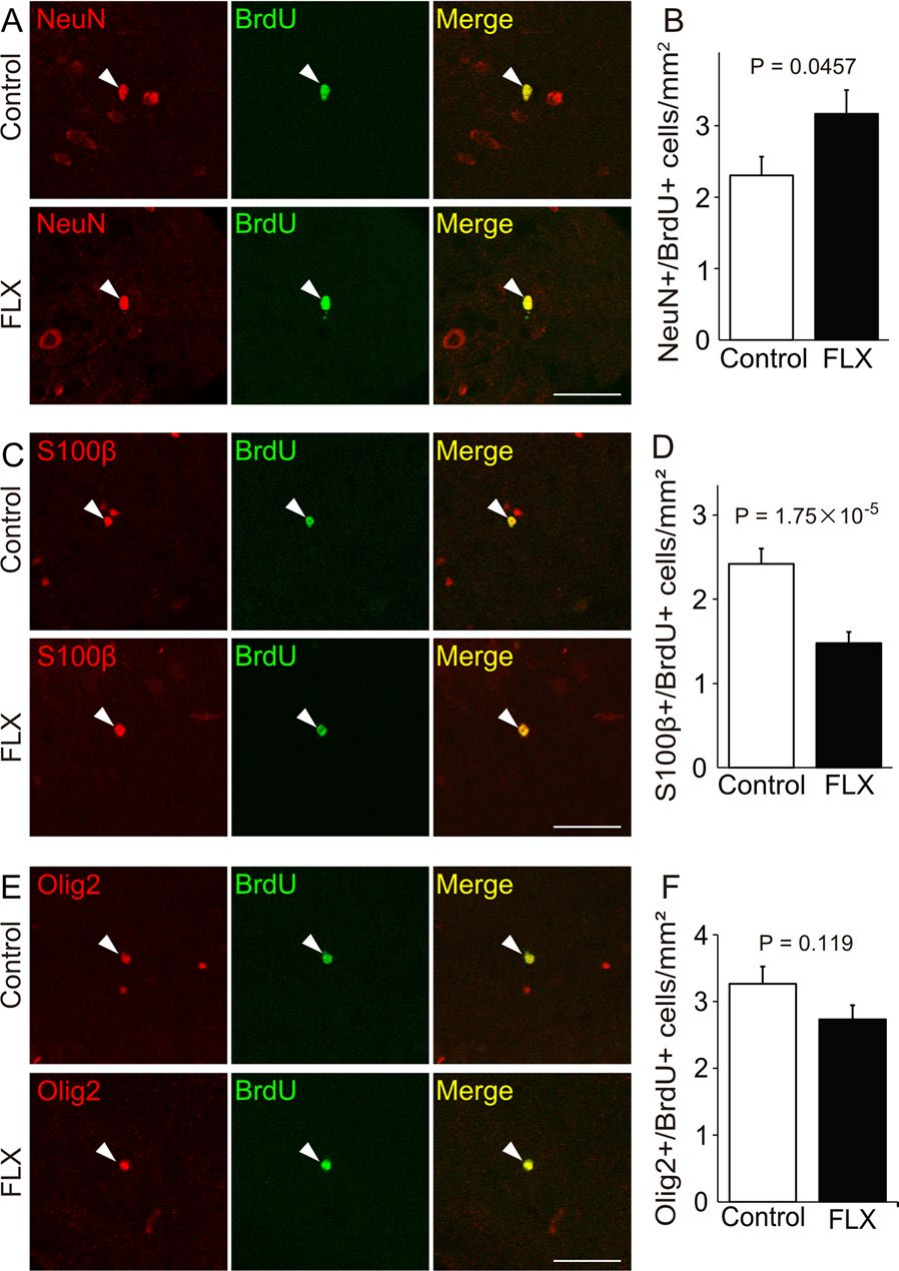
Changes of the numbers of newly-generated cells in the hypothalamus of adult mice. **A**, **B** Images of the neuron marker, NeuN (green) and the proliferating cell marker, BrdU (red), double-positive cells. The quantification of the number of NeuN/BrdU-positive cells is shown. **C**, **D** Images of the astrocyte marker, S100β (green) and BrdU (red) double-positive cells. The quantification of the number of S100β/BrdU-positive cells is shown. (E, F) Images of the oligodendrocyte marker, Olig2 (green) and BrdU (red) double-positive cells. The quantification of the number of Olig2/BrdU-positive cells is shown (n = 4 mice each). Arrowheads indicate the same cells. Scale bar, 50 μm in **A**, **C**, and **E**. Values (mean ± SD) are analyzed by Student’s t test

**Fig. 3:**
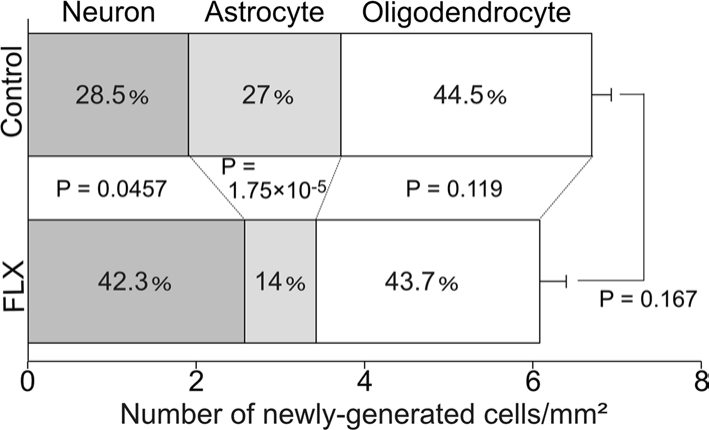
Comparison of total number of newly-generated cells in FLX-treated and control mice. The numbers of new neurons and astrocytes were changed by FLX, but that of oligodendrocytes was not changed. These numbers of neurons, astrocytes, and oligodendrocytes were the same as in Fig. 2. The total number of newly-generated cells was almost unchanged, compared with both groups (n = 4 mice each). Values (mean ± SD) are analyzed by Student’s t test

Next, we determined whether the numbers of newly-produced neurons, astrocytes, and oligodendrocytes, from NSCs were altered by FLX treatments. Contrary to expectations based on decreased densities of hypothalamic NSCs and NPCs in FLX-treated mice, we found that FLX treatments increased the density of new neurons (P = 0.0457), which had the neuron maker NeuN and the cell proliferation marker BrdU, in the hypothalamus (Fig. 2A, B). On the other hand, the density of newly-generated astrocytes, which were the astrocyte marker S100B^+^ and BrdU^+^ cells, was significantly reduced by FLX treatments (P = 1.75 × 10^–5^), compared with controls (Fig. 2C, D). As for oligodendrocytes, which were the oligodendrocyte marker Olig2^+^ and BrdU^+^ cells, there was little change in the numbers between FLX-treated and control mice (P = 0.119) (Fig. 2E, F). The percentage of each cell type (neurons, astrocytes, and oligodendrocytes) in both groups at 4 weeks after vehicle or FLX treatment is shown in Fig. 3. The numbers of total newly-generated cells were not changed between both groups (P = 0.167) (Fig. 3).

## Discussion

Chronic treatments with FLX have been reported to regulate adult neurogenesis: up-regulation in the hippocampus [20], cortex [14], and hypothalamus [27], and down-regulation in the olfactory bulb [21]. These findings suggest that chronic treatments with FLX affect the behaviors of NSCs and NPCs in adult brains. In this study, the experimental data indicate that treatment for 4 weeks with FLX decreased the densities of NSCs and NPCs in the hypothalamus. As mechanisms of this phenomenon, it would be assumed that the expressions of 5-HT receptor subtypes in NSCs and NPCs might be different among them [31–35], and the direction of adult neurogenesis might be determined by the expression levels, balance of receptor subtypes, and the status of intracellular signaling cascades.

In this study, I found that the number of total newly-generated cells was not changed, even though the numbers of NSCs and NPCs were decreased by FLX. The proliferation ability of NSCs and NPCs correlates with the level of nestin expression [30], indicating that the numbers of NSCs and NPCs in the hypothalamus are decreased by FLX. In addition, FLX increased the number of new neurons, decreased that of new astrocytes and did not change that of oligodendrocytes in the hypothalamus. It is conceivable that the increased number of new cells might be due to the longer survival of the cells after division. FLX up-regulates the expression of BDNF in the hypothalamus [27, 36]. BDNF can promote the survival of neurons and oligodendrocytes in the spinal cord [37, 38]. As for astrocytes, FLX upregulates BDNF expression in astrocytes [39], but it remains unclear whether BDNF can increase the survival of astrocytes [40]. Thus, these findings suggest that FLX increases the expression of BDNF, which might promote the survival of new neurons and oligodendrocytes in the hypothalamus. As a result, even though the numbers of NSCs and NPCs are decreased by FLX, the number of total newly-produced cells has not changed. Another possibility is that FLX would promote differentiation into neurons. This action might have reduced the number of NSCs and NPCs, increased that of neurons, and reduced that of astrocytes. However, the findings are the opposite of the findings of previous in vitro evidence [41]. This might reflect the difference between in vitro and in vivo experiments. Further analysis of the effect of FLX on adult neurogenesis in the thalamus is needed, including its relationship to depression and eating disorders (a discussion about depression and eating disorders can be found in Additional file 1).

## Limitations

Although it is relevant to understand how FLX treatment administered to naive control mice affects hypothalamic adult neurogenesis, it is much more important to understand the effect of fluoxetine treatment in depressive models or in animal models of eating disorders. In this study, we performed a quantitative analysis of positive cells and structures by fluorescent intensity. However, since the fluorescence intensity varies slightly between experiments, we made efforts to reduce the variation by experimenting with one sample three times. We also claim from indirect data that new cell numbers have not changed despite FLX treatment. In the future, it will be necessary to perform double staining of BrdU and nuclear staining to directly count new cell numbers.

## Supplementary Information

**Additional file 1: 1.** Rationale and discussion of methodology. **2.** Discussion of hypothalamic neurogenesis, antidepressants, and their relationship to depression and eating disorders.

**Additional file 2.** Antibodies.

## Data Availability

The datasets used during this study are available from the corresponding author upon reasonable request.

## Abbreviations

BrdU: Bromodeoxyuridine
FLX: Fluoxetine
NPC: Neural progenitor cell
NSC: Neural stem cell
PBS: Phosphate-buffered saline
