# Supplementary material for: Change of hypothalamic adult neurogenesis in mice by chronic treatment of fluoxetine
Source: BMC Res Notes. 2022 Feb 16;15:60. doi: 10.1186/s13104-022-05954-z (PMC8848793; doi:10.1186/s13104-022-05954-z)
Supplement: Supplementary file 1 — Additional file 1: 1. Rationale and discussion of methodology. 2. Discussion of hypothalamic neurogenesis, antidepressants, and their relationship to depression and eating disorders. [file 13104_2022_5954_MOESM1_ESM.docx]

1. Rationale and discussion of methodology

In this study, we used 4 male mice per group, and three independent experiments were performed on one mouse, and the average value was used as the value for that mouse. The error of the data due to the experimental operation would be made very small. Further, females have a estrous cycle, and it is already known that fluctuation of female hormones have a significant effect on neurogenesis [1]. In this study, we used male mice to rule out changes in neurogenesis due to changes in female hormones.

In the previous study, mice were treated with FLX by drinking water at the dose of 155mg/L [2]. At 8 weeks of age, C57 male mice weigh about 25-30g (https://www.jax.org/jax-mice-and-services/strain-data-sheet-pages/body-weight-chart-000664#). In addition, our past experiments, which are not unpublished data, have shown that the daily water intake of mice is about 5 ml. From these data, it can be calculated that the dose of FLX to mice is approximately 26-31 mg/kg/day in the study [2]. On the other hand, we treated with FLX at the dose of 15 mg/kg/day. Interestingly, the effects of FLX on behaviors of mice have been found to be highly concentration dependent [3]. Thus, this discrepancy might be due to differences in experimental conditions, most probably the difference in the dose of FLX in 26-31 mg/kg/day in Sachs et al. (2014) vs. 15 mg/kg/day in our study.

2. Discussion of hypothalamic neurogenesis, antidepressants, and their relationship to depression and eating disorders

There are two categories of eating disorders: bulimia nervosa and anorexia nervosa. Bulimia nervosa is an eating disorder characterized by binge eating followed by purging. SSRI is used for treating bulimia nervosa, but its effect would be limited. Rather than those, psychotherapy and psychological interventions have produced better results. On the other hand, anorexia nervosa is characterized by low weight, food restriction, fear of gaining weight and a strong desire to be thin. Antidepressants are not used as sole therapy for anorexia nervosa. Until now, antidepressants have been considered to have little effect on anorexia nervosa, or to have side effects, such as nausea, headache, dizziness, suicidality, and seizures. [4, 5]. But, recently it has been gradually revealed that, among antidepressants, SSRIs have a therapeutic effect [6]. FLX treatment shows significant improvement in body weight and psychiatric symptomatology, such as anxiety, depression, obsession-compulsion, compared with placebo in patients [6]. Controlling neurogenesis in the hypothalamus by drugs or other means may be used to treat eating disorders.

References

1. Jorgensen C, Wang Z. Hormonal regulation of mammalian adult neurogenesis: a multifaceted mechanism. Biomolecules. 2020;10:E1151.

2. Sachs BD, Caron MG. Chronic fluoxetine increases extra-hippocampal neurogenesis in adult mice. Int J Neuropsychopharmacol. 2014;18.

3. Kobayashi K, Ikeda Y, Suzuki H. Behavioral destabilization induced by the selective serotonin reuptake inhibitor fluoxetine. Mol Brain. 2011;4:12.

4. Claudino AM, Hay P, Lima MS, Bacaltchuk J, Schmidt U, Treasure J. Antidepressants for anorexia nervosa. Cochrane Database Syst Rev. 2006;:CD004365.

5. Bulik CM, Berkman ND, Brownley KA, Sedway JA, Lohr KN. Anorexia nervosa treatment: a systematic review of randomized controlled trials. Int J Eat Disord. 2007;40:310–20.

6. Marvanova M, Gramith K. Role of antidepressants in the treatment of adults with anorexia nervosa. Ment Health Clin. 2018;8:127–37.
