# Supplementary material for: Change of hypothalamic adult neurogenesis in mice by chronic treatment of fluoxetine
Source: BMC Res Notes. 2022 Feb 16;15:60. doi: 10.1186/s13104-022-05954-z (PMC8848793; doi:10.1186/s13104-022-05954-z)
Supplement: Supplementary file 2 — Additional file 2. Antibodies. [file 13104_2022_5954_MOESM2_ESM.docx]

***Antibodies***

Primary antibodies

rat anti-BrdU (1:70, abcam, Cambridge, UK)

mouse anti-Nestin (1:200, chemicon, Temecula, CA)

rabbit anti-Ki67 (1:200, abcam)

mouse anti-NeuN (1:200, chemicon)

mouse anti-S100B (1:2000, Sigma)

rabbit anti-Olig2 (1:100, Immuno-Biological Laboratories, Gunma, Japan)

Secondary antibodies

Alexa 488 labeled-goat anti-mouse IgG (1:200, Life Technology, Carlsbad, CA)

Alexa 488 labeled-goat anti-rabbit IgG (1:200, Life Technology)

Alexa 594 labeled-goat anti-rabbit IgG (1:200, Life Technology)

Alexa 594 labeled-goat anti-rat IgG (1:200, Life Technology).
